# Enhancing Viability of *Lactobacillus rhamnosus* GG and Total Polyphenol Content in Fermented Black Goji Berry Beverage Through Calcium–Alginate Encapsulation with Hydrocolloids

**DOI:** 10.3390/foods14030518

**Published:** 2025-02-06

**Authors:** Charoonsri Chusak, Vernabelle Balmori, Kritmongkhon Kamonsuwan, Phim on Suklaew, Sirichai Adisakwattana

**Affiliations:** 1 Center of Excellence in Phytochemical and Functional Food for Clinical Nutrition, Department of Nutrition and Dietetics, Faculty of Allied Health Sciences, Chulalongkorn University, Bangkok 10330, Thailand; charoonsri.c@chula.ac.th (C.C.); 6278303837@student.chula.ac.th (K.K.); sirichai.a@chula.ac.th (S.A.); 2 Department of Food Science and Technology, Southern Leyte State University, Southern Leyte 6606, Philippines; balmori@southernleytestateu.edu.ph; 3 Department of Home Economics, Faculty of Agriculture, Kasetsart University, Bangkok 10900, Thailand

**Keywords:** *Lactobacillus rhamnosus* GG, black goji berry, encapsulation, simulated digestion, hydrocolloids

## Abstract

Encapsulation techniques play a crucial role in enhancing the stability and viability of probiotics in functional foods. This study investigates the efficacy of calcium–alginate encapsulation, combined with hydrocolloids such as carrageenan, agar, and gelatin, in improving the survival of *Lactobacillus rhamnosus* GG (LGG) and stabilizing the total phenolic content (TPC) in fermented black goji berry beverages. The results revealed that 1.5% alginate encapsulation, combined with 1% carrageenan, agar, or gelatin and 5% calcium, significantly enhanced the LGG viability and increased the TPC content in the fermented black goji berry beads when compared to calcium–alginate encapsulation alone. Fourier Transform Infrared Spectroscopy (FTIR) confirmed the successful incorporation and interaction of hydrocolloids within the encapsulation matrix. Among the formulations, calcium–alginate–gelatin beads exhibited the highest LGG survival rates after simulated gastric and intestinal digestion. Notably, calcium–alginate beads containing carrageenan preserved LGG viability during simulated gastric and intestinal conditions when co-digested with all tested milk types (high carbohydrate, high protein, and high fat). Co-ingestion with these milk types further improved TPC retention in all bead formulations, as the macronutrients in milk provided protective effects, stabilizing the encapsulated polyphenols and minimizing their degradation during simulated gastric and intestinal digestion. This study highlights the potential of calcium–alginate encapsulation, integrated with hydrocolloids such as carrageenan, agar, or gelatin, to improve probiotic viability and polyphenol stability, offering promising applications for enhancing the functional properties of non-dairy fermented beverages.

## 1. Introduction

The functional food industry has increasingly focused on developing probiotic-based products due to their well-recognized gut health benefits [1]. Traditionally, most probiotic beverages have been derived from dairy-based ingredients, which limits their appeal to consumers who are lactose intolerant, allergic to milk proteins, or adhere to vegetarian and vegan diets [2]. This has driven the demand for non-dairy alternatives, such as fermented fruit and vegetable juices, kombucha, and plant-based milks, including almond, soy, and oat milks [3]. Fruit and vegetable beverages are rich in minerals, vitamins, antioxidants, and phytochemicals, making them an attractive alternative to dairy products. When fermented with probiotics, these beverages not only retain their nutritional properties but may also exhibit enhanced functional characteristics, such as increased antioxidant activity, appealing to health-conscious consumers [2,4].

Probiotic strains, particularly *Lactobacillus* and *Bifidobacterium*, are widely incorporated into food products for their benefits on digestion, gut health, and immune function [5]. During fermentation, probiotics can biotransform organic acids and phenolic compounds, improving the sensory quality and nutritional properties, including increased short-chain fatty acid (SCFA) production and enhanced biological activity [6,7]. For instance, the fermentation of five pomelo cultivars with *Lacticaseibacillus paracasei* significantly increased the lactic acid bacteria counts, SCFA levels, polyphenols, and flavonoids, leading to enhanced antioxidant activity, bile acid binding, cholesterol micellization disruption, and the inhibition of pancreatic lipase activity [8]. Similarly, Gac juice fermented with *L. paracasei* CASEI 431 exhibited improved probiotic viability, antioxidant activity, and altered volatile compounds, along with higher organic acid and β-carotene contents, positioning it as a promising non-dairy probiotic option [9]. Furthermore, fermented mulberry juice with *L. plantarum* demonstrated increased anthocyanin and phenolic contents, as well as improved antioxidant properties [10]. Despite these advantages, probiotics in juice-based products often struggle to survive the harsh gastrointestinal (GI) conditions, which compromise their viability and health benefits [4]. As a result, current research has focused on techniques to protect probiotics and preserve antioxidants during digestion [4].

Encapsulation has emerged as an effective solution for enhancing the stability, bioavailability, and viability of probiotics and bioactive compounds under harsh GI conditions [11,12,13]. Alginate-based hydrogels are particularly advantageous for encapsulation due to their biocompatibility, gel-forming ability, non-toxicity, and resistance to stomach acidity [13]. When combined with calcium, alginate forms a gel that shields probiotics, allowing for targeted release in the intestines [14]. Encapsulation can also protect antioxidants from degradation during digestion, preserving their bioavailability and functional properties. Additionally, materials such as chitosan, carrageenan, agar, and pectin are often used alongside alginate to further enhance the stability and efficacy of probiotics and bioactive compounds [15].

Black goji berry (*Lycium ruthenicum*), a traditional medicinal food in the *Solanaceae* family, is notable for its high concentrations of polyphenols, anthocyanins, and essential nutrients [16]. It contains elevated levels of cyanidin-3-glucoside, which is higher than in other anthocyanin-rich berries [16,17]. The anthocyanins in black goji berries exhibit potent antioxidant, anti-inflammatory, antilipidemic, and prebiotic-like properties [16,18]. A study by Kamonsuwan et al. [19] demonstrated that *Lactobacillus rhamnosus* GG (LGG) fermentation of black goji berry (BGB) juice significantly enhanced probiotic viability, lactic acid production, and flavonoid content while improving anti-hyperglycemic and anti-hyperlipidemic properties.

With the growing interest in functional fermented beverages that combine probiotics and bioactive compounds, this study focused on developing calcium–alginate encapsulation systems. These systems, incorporating various hydrocolloids, were designed to enhance LGG viability and antioxidant stability in fermented black goji berry (BGB) juice during in vitro gastrointestinal digestion. Additionally, the study explored the application of encapsulated beads in co-digestion with different types of milk, hypothesizing that milk proteins and fats may further protect probiotics and enhance their survival during GI transit.

## 2. Materials and Methods

### 2.1. Chemicals

Black goji berries were sourced from a reputable Chinese dispensary (Chin Heng Huat, Bangkok, Thailand). The probiotic strain *Lactobacillus rhamnosus* GG (LGG) was obtained in freeze-dried direct vat set (DVS) format from Chr. Hansen A/S (Horsholm, Denmark). Gelatin (bovine skin-derived), carrageenan (extracted from *Gracilaria confervoides* seaweed), and agar were purchased from Krungthepchemi Co., Ltd. (Bangkok, Thailand). Reagents, including Folin–Ciocalteu reagent, 2,4,6-tripyridyl-s-triazine (TPTZ), porcine pepsin, α-amylase (Type VI-B, porcine pancreas), and pancreatin, were sourced from Sigma-Aldrich Chemical (St. Louis, MO, USA). *Lactobacillus* MRS agar was obtained from Himedia (Thane, India), while amyloglucosidase (from *Aspergillus niger*) was purchased from Roche Diagnostics (Indianapolis, IN, USA). Sodium alginate, gelatin, carrageenan, and calcium chloride were procured from Nerdy Gummy (Bangkok, Thailand).

### 2.2. Preparation of Fermented Black Goji Berry (BGB) Beverage

To prepare the BGB powder, five grams of dried black goji berry fruit was boiled in 100 mL of distilled water at 95–100 °C for 60 min. The resulting aqueous solution was filtered using Whatman No. 1 filter paper and freeze-dried (Hong Ta Enterprise, Samut Prakarn, Thailand) to obtain the powder, which was stored at −20 °C prior to fermentation. After pasteurizing a mixture of BGB powder (10% *w*/*v*) and sugar (5% *w*/*v*) at 65 °C for 30 min, freeze-dried granules of *LGG* were added to achieve a final bacterial concentration of 7 log CFU/mL [19]. The mixture was gently stirred for 30 min and then allowed to ferment at 37 °C for 24 h in a glass container under anaerobic conditions. The fermented beverage was subsequently stored at −20 °C until further analysis.

### 2.3. Encapsulation Process

The encapsulation process was adapted from previous reports with slight modifications [20]. The formulations included sodium alginate (Ag) combined with the fermented BGB beverage, sodium alginate with carrageenan (Ag + C), sodium alginate with agar (Ag + A), and sodium alginate with gelatin (Ag + G). Sodium alginate solutions (1.2% and 1.5% *w*/*w*) were prepared by dissolving alginate in sterile distilled water at 60 °C under magnetic stirring for 60 min until a homogeneous solution was achieved. Various quantities (0.25, 0.5, and 1 g) of carrageenan, agar, or gelatin were added to 100 g of the sodium alginate solution. Then, 10 mL of the 10% (*w*/*v*) fermented BGB beverage was incorporated, and the mixture was gently stirred to ensure homogeneity and eliminate air bubbles.

**Figure 1:**
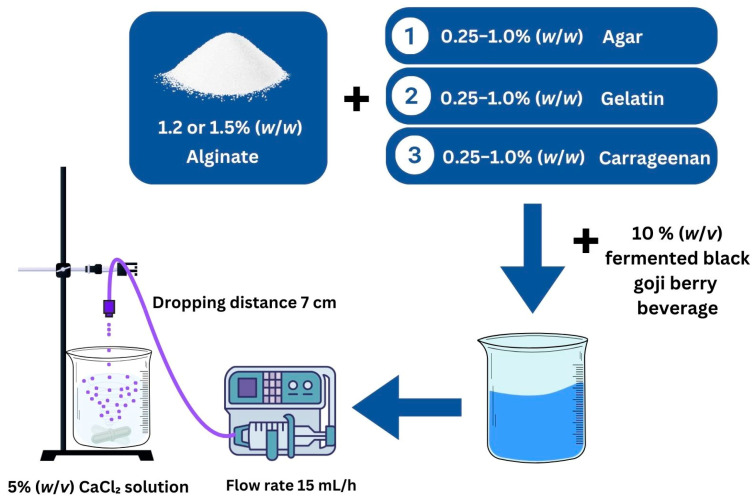
A schematic representation of the encapsulation process for fermented black goji berry in a calcium–alginate system with hydrocolloids.

Encapsulated beads were produced using a syringe pump (Aitecs, Vilnius, Lithuania), which delivered the mixture at a rate of 15 mL/h from a height of 7 cm into a beaker containing sterile CaCl_2_ (5% *w*/*v*). The beads formed instantaneously and were stirred magnetically for 30 min to promote gel formation, as illustrated in the schematic representation of the encapsulation process shown in Figure 1. After rinsing with sterile distilled water, the beads were stored at 4 °C until further analysis.

### 2.4. Determination of Lactic Acid Bacteria

To assess the bacterial count post-encapsulation, the microbial analysis was conducted in accordance with U.S. Food and Drug Administration (U.S. FDA) guidelines using the aerobic plate count method [21]. One gram of the encapsulated bead samples was diluted in 9 mL of a 0.1% (*w*/*v*) sterile peptone solution and allowed to stand for 10 min to facilitate dissolution. The resulting dilutions were agitated and then plated onto MRS agar using a pour plate technique. After incubating at 37 °C for 48 h in an anaerobic environment, plates with up to 250 colonies were counted using a colony counter. The results were reported as colony-forming units per gram (cfu/g sample).

### 2.5. Determination of Total Phenolic Content

Encapsulated beads (2 g) were dissolved in 5 mL of 5% (*w*/*v*) sodium citrate until completely dissolved. The resulting solutions were subsequently analyzed for their total phenolic content (TPC) and antioxidant activity. The total phenolic content was determined using the Folin–Ciocalteu reagent, as described by Chusak et al. [22]. Fifty microliters of the dissolved beads were mixed with 50 μL of 10% (*v*/*v*) Folin–Ciocalteu reagent. After a 10 min incubation at room temperature, 50 μL of 10% (*w*/*v*) Na_2_CO_3_ was added, and the mixture was allowed to stand for 30 min in the dark. The absorbance was measured at 760 nm, using gallic acid as a standard. The encapsulation efficiency (EE%) was calculated as the percentage of the ratio between the TPC of encapsulated beads in the final product and the initial TPC to be encapsulated.

### 2.6. Characterization of Encapsulation Beads

#### 2.6.1. Fourier Transform Infrared Spectroscopy (FT-IR)

Fourier Transform Infrared (FT-IR) Spectroscopy was employed to characterize the interactions among bead components. FT-IR spectra were recorded using a spectrometer (Alpha II, Bruker, Billerica, MA, USA), with 4 scans collected across a range of 675 to 4000 cm^−1^ at a resolution of 4 cm^−1^. Control reagents, including sodium alginate, carrageenan, gelatin, and agar, as well as the encapsulated beads, were dried prior to the measurements. The peaks in the FT-IR spectra were interpreted to assess the chemical interactions.

#### 2.6.2. Scanning Electron Microscopy (SEM)

The morphological properties of the encapsulated beads were analyzed using Scanning Electron Microscopy (SEM) (JSM-IT300LV, JEOL Co., Tokyo, Japan) at an acceleration voltage of 30 kV. Samples were prepared by drying them with a Critical Point Dryer (Leica EM CPD300, Leica, Wetzlar, Germany) before SEM analysis.

### 2.7. Survival of Microorganisms After In Vitro Simulated Gastric and Intestinal Fluids

The survival of microorganisms was evaluated using a modified method adapted from that of Mirmazloum et al. [23]. Two-gram samples of the mixture solution and encapsulated beads were added to tubes containing 2 mL of simulated gastric juice (6 M HCl with 0.2% *w*/*v* NaCl, pH 2.5) and incubated at 37 °C for 30 min. Subsequently, 10 mL of simulated intestinal juice (5 mM phosphate buffer, pH 7) containing 5% bile salt was introduced, and the tubes were incubated at 37 °C for 180 min.

Following incubation, the beads were retrieved, rinsed with sterile distilled water, and analyzed for their lactic acid bacteria counts and total phenolic content (TPCs). Beads prepared with alginate and hydrocolloids, without the fermented beverage, served as the control.

### 2.8. Survival of Microorganisms After Co-Ingestion with Different Types of Milk

To evaluate the effects of different macronutrient ratios, three types of milk were prepared: high fat (HF), providing 53% of energy from fat; high protein (HP), providing 39% of energy from protein; and high carbohydrate (HC), providing 72% of energy from carbohydrates. The digestion process followed a modified version of the methodology by Suklaew et al. [24]. Initially, 2 g of encapsulated beads was combined with 5 mL of each milk type. Digestion began with the addition of 1 mL of α-amylase (250 U/mL in 0.2 M carbonate buffer, pH 7), followed by a 1 min incubation. Subsequently, 5 mL of porcine pepsin solution (3200 U/mL in 0.02 N HCl, pH 2) was introduced, and the mixture was incubated at 37 °C for 30 min in a water bath shaker set to 100 rpm. To neutralize the gastric phase, 5 mL of 0.02 N NaOH and 20 mL of 0.2 M sodium acetate buffer (pH 6) were added. The intestinal phase was initiated by incorporating 5 mL of 5% bile extract and 5 mL of a mixture containing pancreatin (2 mg/mL) and amyloglucosidase (28 U/mL) prepared in sodium acetate buffer (pH 6). This mixture was incubated at 37 °C for 180 min. Following the digestion process, the beads were collected, rinsed with sterile distilled water, and subjected to analyses for their lactic acid bacteria counts and total phenolic content (TPCs).

### 2.9. Statistical Analysis

The results were expressed as the mean ± standard error of the mean (SEM). Statistical analysis was performed using one-way Analysis of Variance (ANOVA), followed by Duncan’s post hoc test to identify significant differences between samples. All analyses were conducted using SPSS software (version 22).

## 3. Results and Discussion

### 3.1. Effects of Hydrocolloids on Lactic Acid Bacteria Viability in Alginate Beads

The impacts of carrageenan (C), agar (A), and gelatin (G) on the viability of lactic acid bacteria encapsulated within 1.2% alginate (Ag) were evaluated, as summarized in Table 1. The encapsulation of 1.2% Ag alone resulted in a bacterial count of 119.8 × 10^3^ CFU/g. The addition of carrageenan slightly reduced the bacterial viability, with the 0.5% carrageenan formulation showing the highest count, while the 1% carrageenan formulation exhibited the lowest count. Agar significantly decreased the bacterial viability across all concentrations, with the most pronounced reduction observed at 1% A. Gelatin also reduced the bacterial counts, although 0.5% G demonstrated higher viability compared to 1% G. These results indicate that the inclusion of carrageenan, agar, or gelatin in 1.2% alginate does not enhance bacterial viability and may adversely affect it.

**Figure 2:**
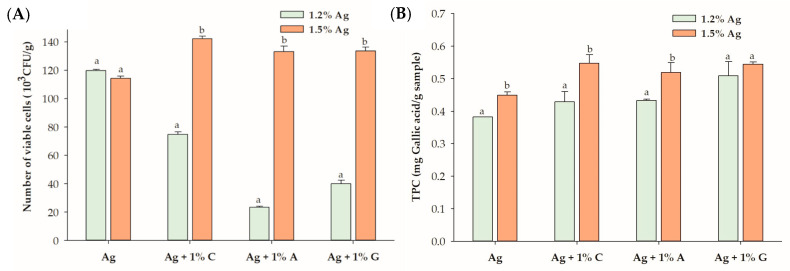
(**A**) Viability of *Lactobacillus rhamnosus* GG (LGG) and (**B**) total polyphenol content (TPC) in the encapsulated beads, comparing 1.2% alginate (Ag) and 1.5% Ag with 1% hydrocolloids. Data are expressed as the mean ± standard error of the mean (SEM) (n = 3). Different letters indicate significant differences between 1.2% Ag and 1.5% Ag at *p* < 0.05. C: carrageenan; A: agar; G: gelatin.

At 1.5% alginate (Ag), which alone demonstrated a cell viability of 114.3 × 10^3^ CFU/g, the addition of 1% carrageenan (C), agar (A), or gelatin (G) significantly enhanced the viability of lactic acid bacteria within the encapsulated beads compared to alginate alone. Comparing the highest concentrations of carrageenan (1% C), agar (1% A), and gelatin (1% G) at 1.5% and 1.2% alginate revealed substantial improvements in the cell viability with increased alginate concentration (Figure 2). Specifically, carrageenan increased the viability by 1.9-fold, agar demonstrated a 5.7-fold enhancement, and gelatin exhibited a 3.3-fold increase. These findings suggest that alginate-based beads effectively encapsulate LGG in fermented black goji berry formulations. However, at lower alginate concentrations, the inclusion of carrageenan, agar, or gelatin may interfere with the gelation process by competing for water and calcium ions, disrupting cross-link formation, and compromising gel stability. This interference could result in a porous bead matrix that is less effective in retaining and protecting probiotics. In contrast, increasing the alginate concentration enhances the hydrocolloid matrix’s structural integrity, improving its protective capacity and probiotic viability, particularly when combined with hydrocolloids such as carrageenan, gelatin, or agar. As noted by Banerjee et al. [25], higher alginate concentrations reduce surface pore sizes, creating a denser and more compact matrix that enhances encapsulation efficiency. Additionally, Lotfipour et al. [26] also suggested that higher alginate concentrations could be attributed to the firmer structure of the beads formed, which may prevent probiotic release during the hardening and coating process.

Evidence suggests that co-encapsulating probiotics with carrageenan, gelatin, and agar enhances encapsulation efficiency [12]. For example, double-network gels combining fish gelatin and sodium alginate significantly improved the encapsulation efficiency of *Bifidobacterium longum* compared to alginate alone [27]. Similarly, the use of sodium alginate/CaCl_2_/KCl with κ-carrageenan enhanced the encapsulation efficiency of *Streptococcus thermophilus* [28]. Furthermore, the probiotic bacterium LPMB001, co-encapsulated with alginate and agar in an oil-in-water emulsion, formed stable microcapsules via calcium chloride cross-linking, achieving high encapsulation efficiency [29]. The present study is the first to demonstrate that co-encapsulating calcium–alginate with carrageenan, gelatin, and agar improves the loading efficiency of LGG-fermented black goji berry formulations compared to calcium–alginate alone. The inclusion of these additional polymers likely creates a more robust and stable matrix, enhancing encapsulation efficiency and providing better protection under external conditions. The formation of calcium–alginate/κC hydrogels through the interaction of calcium–alginate and carrageenan results in tighter gel structures, improved ionic cross-linking, and greater stability, further improving probiotic encapsulation efficiency.

### 3.2. Effects of Hydrocolloids on Total Phenolic Content in Alginate Beads

The impact of carrageenan, agar, and gelatin on the TPC of the probiotic black goji berry beverage was assessed at 1.2% and 1.5% alginate concentrations (Table 1). At 1.2% alginate, only the combination with 1% agar significantly increased the TPC from 0.382 mg gallic acid/g (for alginate alone) to 0.509 mg gallic acid/g (*p* < 0.05). In contrast, neither carrageenan nor gelatin significantly improved the TPC at this concentration, likely due to the weaker gel structure formed by the lower alginate concentration, which may hinder phenolic retention. The change in gel structure is largely influenced by the concentration of hydrocolloid molecules in the junction zone, with a higher concentration resulting in a stronger gel among a three-dimensional structure [30].

When the alginate concentration was increased to 1.5%, significant improvements in the TPC were observed for all three hydrocolloids—1% carrageenan, agar, and gelatin—indicating that higher alginate concentrations facilitate more effective encapsulation. The denser and more cohesive matrix formed at 1.5% alginate likely reduces the pore size and enhances the gel’s barrier properties, thereby minimizing polyphenol loss during encapsulation. At lower alginate concentrations, carrageenan and gelatin may lack sufficient structural support or favorable interactions with the alginate matrix to effectively retain phenolic compounds. However, the denser network formed at 1.5% alginate strengthens the interactions between hydrocolloids and the alginate matrix, enhancing TPC retention [25,31]. The encapsulation efficiency (EE%) of beads was influenced by the concentration of alginate and the types of hydrocolloids used. The EE values ranged from 35.6% to 42.7% for 1.2% alginate and 35.0% to 45.3% for 1.5% alginate. Alginate alone, at both concentrations, exhibited the lowest EE, while higher concentrations of hydrocolloids, particularly gelatin, resulted in significantly improved EE values. These findings highlight the critical role of optimizing the alginate concentration and hydrocolloid combinations to maximize polyphenol retention in encapsulated systems.

### 3.3. Morphology and FTIR of the Encapsulated Beads

**Figure 3:**
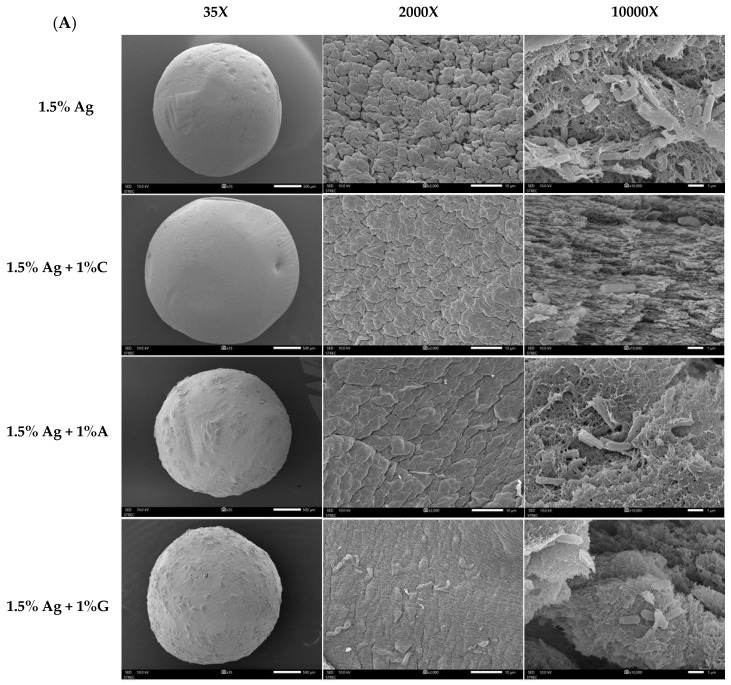

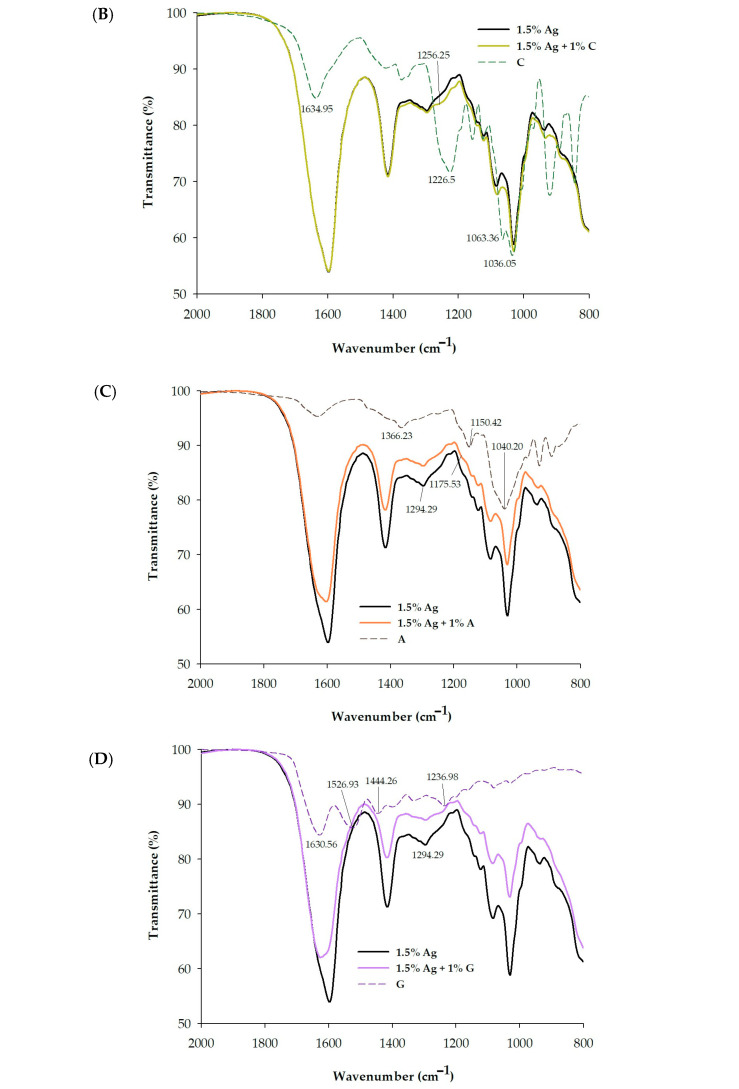
(**A**) Scanning Electron Microscopy (SEM) images of 1.5% Ag (alginate), 1.5% Ag + 1% C (carrageenan), 1.5% Ag + 1% A (agar), and 1.5% Ag + 1% G (gelatin) encapsulated beads. The overall structure is shown at 30× magnification (left), surface morphology at 2000× magnification (middle), and cross-section at 10,000× magnification (right). (**B**) FT-IR spectra of 1.5% Ag, 1.5% Ag + 1% C, and C encapsulated beads. (**C**) FT-IR spectra of 1.5% Ag, 1.5% Ag + 1% A, and A encapsulated beads. (**D**) FT-IR spectra of 1.5% Ag, 1.5% Ag + 1% G, and G encapsulated beads.

Figure 3A presents SEM images of the encapsulated beads, highlighting their surface and cross-sectional features at various magnifications (35×, 2000×, and 10,000×). Although all formulations exhibited a spherical shape, differences in the surface texture and internal structure were evident based on the co-encapsulation materials used. Beads containing 1.5% alginate (Ag) showed a low-density structure with a porous surface and interior. This less compact matrix is likely to result in reduced probiotic retention and weaker encapsulation stability, potentially compromising the protection of the encapsulated bacteria. In comparison, beads made with 1.5% Ag + 1% carrageenan (C) had smoother surfaces and a denser internal structure with smaller pores. These features indicate enhanced gel strength and internal cohesion, making the carrageenan–alginate matrix more effective at retaining probiotics. The 1.5% Ag + 1% gelatin (G) beads showed an elastic matrix with moderate surface improvements [32]. While the surface was smoother than pure alginate beads, it was not as compact as the carrageenan formulation. Internally, the gelatin combination formed a thicker, layered structure, adding flexibility to the gel matrix and offering moderate protection for the encapsulated probiotics. Lastly, beads made with 1.5% Ag + 1% agar (A) exhibited a rigid matrix with a dense surface and a highly compact internal structure with minimal voids. The rigid nature of the alginate–agar combination provides superior mechanical stability, which is advantageous for probiotic retention under adverse conditions [33].

Figure 3B–D present the FTIR spectra of various bead formulations, analyzed to elucidate the molecular interactions between the encapsulating agents and the encapsulated compounds. The characteristic peak for the carboxylate groups (C=O stretching) of alginate is typically found around 1600–1650 cm^−1^ [34]. When calcium ions are added, this peak shifts to a lower wavenumber, often around 1550–1600 cm^−1^, indicating that the carboxylate groups interact with calcium ions, leading to ionic cross-linking [35,36]. Carrageenan exhibits distinct sulfate-related peaks, with strong absorption bands in the 1210–1260 cm⁻¹ region (due to S=O of sulfate esters) and in the 1010–1080 cm^−1^ region (ascribed to glycosidic linkages) [37]. The FTIR analysis reveals that both the alginate–calcium complex and the alginate–calcium–carrageenan complex exhibit the same peak at 1596.64 cm^−1^, indicating that the ionic interactions between alginate and calcium remain intact even with the incorporation of carrageenan. This suggests that carrageenan does not disrupt the existing ionic cross-linking of the alginate–calcium matrix [38]. While the peak position remains unchanged, additional FTIR data, including shifts in the 1250–900 cm^−1^ range and reductions in peak intensity, suggest that carrageenan contributes to a more complex and cohesive gel network through hydrogen bonding [28]. This modified structure provides enhanced mechanical stability and creates a more effective environment for encapsulating both probiotics and polyphenols, ultimately improving their retention and viability.

FTIR analysis of gelatin revealed key spectral features indicative of its molecular structure. The amide I band, located around 1650–1700 cm^−1^, corresponds to C=O stretching in peptide bonds, while the amide II band, found at 1530–1560 cm^−1^, reflects N-H bending and C-N stretching (Figure 3B). The fingerprint region between 1000 and 1200 cm^−1^ includes C-O stretching vibrations, serving as a unique identifier for gelatin types [30]. In contrast, FTIR analysis of agar, as illustrated in Figure 3C, shows distinctive peaks that reflect its polysaccharide structure, particularly absorption bands around 1200–950 cm^−1^ associated with C-O stretching vibrations in the agarose backbone [39]. The FTIR analysis also reveals significant interactions between gelatin or agar and the alginate–calcium complex. The observed shift from 1596 cm^−1^ in the alginate–calcium complex to 1625 cm^−1^ in the alginate–calcium–gelatin or agar composite suggests that while ionic cross-linking between alginate and calcium remains strong, the addition of gelatin or agar alters these interactions. This modification likely arises from hydrogen bonding between the amine groups of gelatin (or corresponding functional groups in agar) and the carboxylate groups of alginate, enhancing the structural integrity of the gel.

Additionally, a notable decrease in peak intensity within the 1550 to 800 cm^−1^ range indicates that these interactions contribute to a more cohesive network. This reduction in intensity suggests stabilized molecular vibrations within the gel matrix. These synergistic effects not only enhance the mechanical properties and stability of the hydrogel network but also significantly improve the encapsulation efficiency of both probiotics and polyphenols.

### 3.4. Survival Rate of Encapsulated Beads After In Vitro Simulated Gastric and Intestinal Digestion

In the black goji berry fermented juice, the LGG strain initially exhibited a concentration of approximately 10^7^ CFU/mL. However, after being exposure to in vitro simulated gastric and intestinal fluids for 180 min, no viable lactic acid bacteria were detected in the black goji berry fermented beverage. These results are consistent with previous studies reporting significant reductions or complete loss of viable *Lactobacillus* bacteria in fermented probiotic juices after gastrointestinal digestion [40,41]. The survival of *Lactobacillus* is compromised during this process due to the harsh acidic conditions in the stomach and exposure to bile salts and digestive enzymes in the intestines, which adversely affect probiotic viability [42]. Therefore, encapsulation techniques have been shown to enhance the gastric and intestinal tolerance of probiotic bacteria [12].

**Figure 4:**
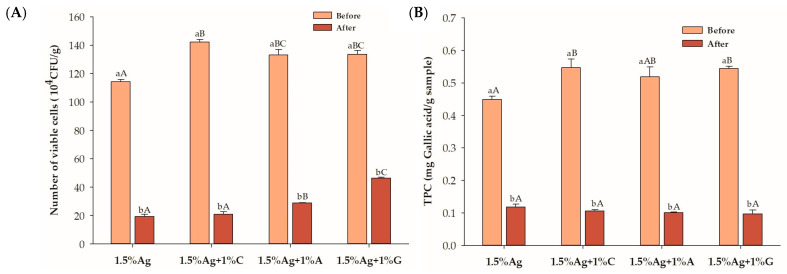
(**A**) Viability of *Lactobacillus rhamnosus* GG (LGG) and (**B**) total polyphenol content (TPC) in the encapsulated beads before and after 180 min of in vitro simulated gastric and intestinal digestion. Data are expressed as the mean ± SEM (n = 3). Different letters indicate statistically significant differences at *p* < 0.05. Uppercase letters (A–C) indicate comparisons between 1.5% Ag, 1.5% Ag + 1% C, 1.5% Ag + 1% A, and 1.5% Ag + 1% G before or after digestion. Lowercase letters (a,b) represent comparisons between before and after digestion within the same sample.

Figure 4A presents the viable cell counts of LGG in beads following a 180 min post-digestion period (refer to Figure 4). Beads formulated with 1.5% alginate (Ag) and 1.5% Ag + 1% carrageenan (C) showed a reduction in LGG viability after digestion. In contrast, formulations containing 1.5% Ag combined with 1% gelatin (G) exhibited the highest viable counts, followed closely by those with 1.5% Ag and 1% agar (A). The improved viability in gelatin- and agar-containing beads can be attributed to their ability to strengthen the hydrogel network and create additional protective barriers against the disturbing gastric and intestinal conditions. Gelatin enhances encapsulation through hydrogen bonding with alginate, contributing to improved gel flexibility and resilience, which shields probiotics during digestion [14]. Similarly, agar forms a more rigid network that likely provides mechanical support, preventing excessive release of probiotics during digestion [43]. In comparison, carrageenan’s softer gel structure may be less effective in maintaining the integrity of the beads under digestive stress, leading to lower viable counts [44]. The enhanced mechanical properties of gelatin- and agar-based matrices likely improved the survival of probiotics compared to the carrageenan-based formulation. These findings indicate that beads retaining viable probiotics after 180 min of digestion can effectively deliver them to the large intestine, a favorable environment for their release and activity. The release of probiotics, such as LGG, in the large intestine may positively impact gut health, as this environment supports their proliferation and ability to modulate the gut microbiota [45]. This modulation can lead to improved gut barrier function and the inhibition of pathogenic bacteria, ultimately contributing to enhanced gastrointestinal health and immune modulation [45].

The TPCs of all formulations decreased after simulated digestion, indicating partial degradation or the release of polyphenols during the gastrointestinal process (Figure 4B). Although some of the TPC was lost, its retention within the beads may be advantageous if released in the large intestine, where it can act as a prebiotic to enhance probiotic survival. These phenolic compounds may support the viability and activity of probiotics, promoting a healthier gut microbiome and improved gut health [46]. Further studies are needed to clarify the effects of the TPC on gut microbiota modulation in an in vitro colonic fermentation model.

### 3.5. Viability of LGG and TPC in Encapsulated Beads Co-Ingested with Different Types of Milk

This study evaluates the stability and viability of encapsulated BGB beads co-ingested with different milk types, focusing on their ability to protect LGG probiotics and maintain the TPC during digestion. Hydrocolloid-based beads (alginate, carrageenan, agar, and gelatin) enhance the survival of probiotics and polyphenols. The study compares the effects of high-carbohydrate, high-protein, and high-fat milks on probiotic survival and TPC retention in these bead formulations.

**Figure 5:**
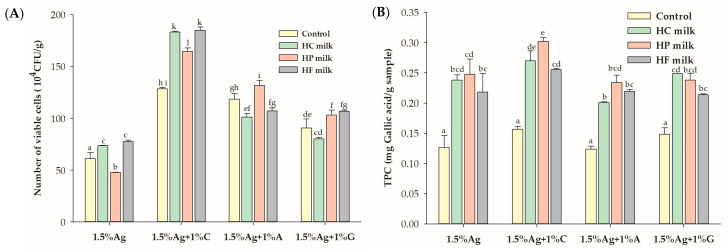
Effect of co-ingestion with different milk types (high carbohydrate (HC), high protein (HP), and high fat (HF)) on (**A**) the viability of *Lactobacillus rhamnosus* GG (LGG) and (**B**) total polyphenol content (TPC) in the encapsulated beads after in vitro simulated gastric and intestinal digestion. Control refers to beads in water. Data are presented as the mean ± standard error of the mean (SEM) (n = 3). Statistical differences are denoted by different superscript letters (*p* < 0.05).

As shown in Figure 5, after 180 min of digestion, beads containing alginate and carrageenan (Ag + C) exhibited the highest survival of LGG, with a 1.4-fold increase in survival compared to the water matrix (control). Beads made with alginate and agar (Ag + A) enhanced LGG survival only in high-protein milk, while beads with alginate and gelatin (Ag + G) improved survival in both high-protein and high-fat milks. The addition of carrageenan significantly improved probiotic survival in all milk types, emphasizing its protective effect. This increased LGG survival in milk is likely due to several factors: carrageenan strengthens the alginate matrix [47], enhancing stability against gastrointestinal conditions; milk proteins form a protective layer around the beads, reducing exposure to stomach acid and digestive enzymes; and milk fats provide a hydrophobic barrier, further stabilizing the bacteria. Additionally, milk’s nutritional content supports bacterial growth, as demonstrated by studies like that of Ni et al. [48], where sucrose in a milk tea model increased *Lactobacillus plantarum* survival. Together, these factors improve probiotic viability, allowing encapsulated beads to deliver more live probiotics to the large intestine, potentially modulating the gut microbiome and promoting gut health. These results indicate that milk, especially varieties rich in protein and fat, significantly enhances probiotic survival compared to the control, providing an ideal matrix for safeguarding LGG during gastrointestinal transit.

No significant differences in TPC retention were observed across bead formulations when co-ingested with water. However, all bead formulations showed improved TPC retention when co-ingested with milk. This enhancement is likely due to the protective effects of milk’s macronutrients, such as proteins and fats, which stabilize the encapsulated phenolic compounds during digestion. Milk buffers stomach acidity, preventing phenolic degradation and maintaining TPC levels. Milk macronutrients likely stabilize polyphenols through hydrophobic interactions and protein–polyphenol complexation, as supported by similar studies [49]. Previous research has shown that the bioaccessibility of black currant extract (BCE) is lowest when co-ingested with water compared to starch, oil, or protein, emphasizing the protective role of macronutrients on polyphenols during digestion [50]. Milk proteins, through hydrophobic interactions, form complexes with polyphenols, reducing their degradation and enhancing their stability [51]. The retained TPC in the beads, when transported to the large intestine, may act as a prebiotic, potentially fostering the growth of beneficial gut microbiota and supporting gut health.

## 4. Conclusions

Calcium–alginate (1.5%) encapsulation combined with hydrocolloids (1%) such as carrageenan, agar, and gelatin, significantly enhanced LGG viability and improved TPC stability in fermented black goji berry beverages compared to calcium–alginate encapsulation alone or calcium–alginate (1.2%) encapsulation with or without hydrocolloids. Beads with alginate (1.5%) and gelatin (1%) exhibited the highest LGG survival following simulated digestion, while those with carrageenan preserved LGG viability across all milk types (high carbohydrate, high protein, and high fat). Co-digestion with milk further enhanced TPC retention, attributed to the macronutrient stabilization of polyphenols. These findings highlight the potential of calcium–alginate–hydrocolloid encapsulation in enhancing both probiotic viability and polyphenol stability in non-dairy fermented beverages.

## Figures and Tables

**Table 1:** Viability of *Lactobacillus rhamnosus* GG (LGG) and total polyphenol content (TPC) in fermented black goji berry beverage beads.

Sample	Number of Viable Cells (10^3^ CFU/g)	TPC (mg Gallic Acid/g)
1.2% Ag	119.8 ± 0.78 ^a^	0.382 ± 0.000 ^a,b^
1.2% Ag + 0.25% C	98.7 ± 1.22 ^b^	0.372 ± 0.031 ^a^
1.2% Ag + 0.5% C	109.0 ± 8.56 ^a,b^	0.367 ± 0.040 ^a^
1.2% Ag + 1% C	74.8 ± 1.85 ^c^	0.429 ± 0.013 ^a,b,c^
1.2% Ag + 0.25% A	41.3 ± 4.92 ^d^	0.387 ± 0.003 ^a,b^
1.2% Ag + 0.5% A	27.0 ± 2.01 ^e^	0.348 ± 0.034 ^a^
1.2% Ag + 1% A	23.5 ± 0.54 ^e^	0.433 ± 0.004 ^a,b,c^
1.2% Ag + 0.25% G	40.2 ± 5.43 ^d^	0.413 ± 0.023 ^a,b^
1.2% Ag + 0.5% G	73.5 ± 2.64 ^c^	0.460 ± 0.003 ^b,c^
1.2% Ag + 1% G	40.0 ± 2.43 ^d^	0.509 ± 0.043 ^c^
1.5% Ag	114.3 ± 1.75 ^a,c^	0.449 ± 0.010 ^a^
1.5% Ag + 0.25% C	117.5 ± 3.01 ^a,c^	0.469 ± 0.012 ^a,b^
1.5% Ag + 0.5% C	126.3 ± 4.70 ^c,d^	0.486 ± 0.016 ^a,b^
1.5% Ag + 1% C	142.3 ± 1.85 ^e^	0.547 ± 0.026 ^b^
1.5% Ag + 0.25% A	80.4 ± 5.07 ^b^	0.446 ± 0.041 ^a^
1.5% Ag + 0.5% A	116.4 ± 3.10 ^a,c^	0.468 ± 0.017 ^a,b^
1.5% Ag + 1% A	133.1 ± 4.05 ^d,e^	0.532 ± 0.050 ^b^
1.5% Ag + 0.25% G	110.1 ± 4.97 ^a^	0.458 ± 0.007 ^a^
1.5% Ag + 0.5% G	71.5 ± 4.03 ^b^	0.527 ± 0.036 ^a,b^
1.5% Ag + 1% G	133.7 ± 2.66 ^d,e^	0.544 ± 0.007 ^b^

Data are presented as the mean ± standard error of the mean (SEM), n = 3. Different superscript letters denote statistically significant differences within the same alginate (Ag) concentration at *p* < 0.05. C: carrageenan; A: agar; G: gelatin.

## Data Availability

The original contributions presented in this study are included in the article. Further inquiries can be directed to the corresponding author.

## References

[1] Kailasapathy K., Tamang J.P., Kailasapathy K. (2010). Probiotic and prebiotic fermented foods. Fermented Foods and Beverages of the World.

[2] Gomes I.A., Venâncio A., Lima J.P., Freitas-Silva O. (2021). Fruit-based non-dairy beverage: A new approach for probiotics. Adv. Biol. Chem..

[3] Gonçalves G., Weis C., Wolff E., Alves V., Sanches F., Tormen L., Treichek H. (2025). Vegan fermented drinks as an alternative to milk: Trend or challenge?. Food Sci. Eng..

[4] Valero-Cases E., Cerdá-Bernad D., Pastor J.J., Frutos M.J. (2020). Non-dairy fermented beverages as potential carriers to ensure probiotics, prebiotics, and bioactive compounds arrival to the gut and their health benefits. Nutrients.

[5] Das T.K., Pradhan S., Chakrabarti S., Mondal K.C., Ghosh K. (2022). Current status of probiotic and related health benefits. Appl. Food Res..

[6] Wu C., Li T., Qi J., Jiang T., Xu H., Lei H. (2020). Effects of lactic acid fermentation-based biotransformation on phenolic profiles, antioxidant capacity and flavor volatiles of apple juice. LWT-Food Sci. Technol..

[7] Zhong H., Zhao M., Tang J., Deng L., Feng F. (2021). Probiotics-fermented blueberry juices as potential antidiabetic product: Antioxidant, antimicrobial and antidiabetic potentials. J. Sci. Food Agric..

[8] Balmori V., Marnpae M., Chusak C., Kamonsuwan K., Katelakha K., Charoensiddhi S., Adisakwattana S. (2023). Enhancing phytochemical compounds, functional properties, and volatile flavor profiles of pomelo (*Citrus grandis* (L.) Osbeck) juices from different cultivars through fermentation with *Lacticaseibacillus paracasei*. Foods.

[9] Marnpae M., Chusak C., Balmori V., Kamonsuwan K., Dahlan W., Nhujak T., Hamid N., Adisakwattana S. (2022). Probiotic Gac fruit beverage fermented with *Lactobacillus paracasei*: Physiochemical properties, phytochemicals, antioxidant activities, functional properties, and volatile flavor compounds. LWT-Food Sci. Technol..

[10] Kwaw E., Ma Y., Tchabo W., Apaliya M.T., Wu M., Sackey A.S., Xiao L., Tahir H.E. (2018). Effect of *Lactobacillus* strains on phenolic profile, color attributes and antioxidant activities of lactic-acid-fermented mulberry juice. Food Chem..

[11] Heidebach T., Först P., Kulozik U. (2012). Microencapsulation of probiotic cells for food applications. Crit. Rev. Food Sci. Nutr..

[12] Ta L.P., Bujna E., Antal O., Ladányi M., Juhász R., Szécsi A., Kun S., Sudheer S., Gupta V.K., Nguyen Q.D. (2021). Effects of various polysaccharides (alginate, carrageenan, gums, chitosan) and their combination with prebiotic saccharides (resistant starch, lactosucrose, lactulose) on the encapsulation of probiotic bacteria *Lactobacillus casei* 01 strain. Int. J. Biol. Macromol..

[13] Wang X., Gao S., Yun S., Zhang M., Peng L., Li Y., Zhou Y. (2022). Microencapsulating alginate-based polymers for probiotics delivery systems and their application. Pharmaceuticals.

[14] Sun Q., Yin S., He Y., Cao Y., Jiang C. (2023). Biomaterials and encapsulation techniques for probiotics: Current status and future prospects in biomedical applications. Nanomaterials.

[15] Koh W.Y., Lim X.X., Tan T.C., Kobun R., Rasti B. (2022). Encapsulated probiotics: Potential techniques and coating materials for non-dairy food applications. Appl. Sci..

[16] Vidana Gamage G.C., Lim Y.Y., Choo W.S. (2021). Black goji berry anthocyanins: Extraction, stability, health benefits, and applications. ACS Food Sci. Technol..

[17] Liu B., Xu Q., Sun Y. (2020). Black goji berry (*Lycium ruthenicum*) tea has higher phytochemical contents and in vitro antioxidant properties than red goji berry (*Lycium barbarum*) tea. Food Qual. Saf..

[18] Vidović B.B., Milinčić D.D., Marčetić M.D., Djuriš J.D., Ilić T.D., Kostić A.Ž., Pešić M.B. (2022). Health benefits and applications of goji berries in functional food products development: A review. Antioxidants.

[19] Kamonsuwan K., Balmori V., Marnpae M., Chusak C., Thilavech T., Charoensiddhi S., Smid S., Adisakwattana S. (2024). Black Goji Berry (*Lycium ruthenicum*) juice fermented with *Lactobacillus rhamnosus* GG enhances inhibitory activity against dipeptidyl peptidase-IV and key steps of lipid digestion and absorption. Antioxidants.

[20] Pasukamonset P., Kwon O., Adisakwattana S. (2016). Alginate-based encapsulation of polyphenols from *Clitoria ternatea* petal flower extract enhances stability and biological activity under simulated gastrointestinal conditions. Food Hydrocoll..

[21] Maturin L., Peeler J. (2001). BAM: Aerobic Plate Count.

[22] Chusak C., Chanbunyawat P., Chumnumduang P., Chantarasinlapin P., Suantawee T., Adisakwattana S. (2020). Effect of gac fruit (*Momordica cochinchinensis*) powder on in vitro starch digestibility, nutritional quality, textural and sensory characteristics of pasta. LWT-Food Sci. Technol..

[23] Mirmazloum I., Ladányi M., Omran M., Papp V., Ronkainen V.P., Pónya Z., Papp I., Némedi E., Kiss A. (2021). Co-encapsulation of probiotic *Lactobacillus acidophilus* and Reishi medicinal mushroom (*Ganoderma lingzhi*) extract in moist calcium alginate beads. Int. J. Biol. Macromol..

[24] Suklaew P.O., Chusak C., Adisakwattana S. (2020). Physicochemical and functional characteristics of RD43 rice flour and its food application. Foods.

[25] Banerjee S., Tiwade P.B., Sambhav K., Banerjee C., Bhaumik S.K. (2019). Effect of alginate concentration in wastewater nutrient removal using alginate-immobilized microalgae beads: Uptake kinetics and adsorption studies. Biochem. Eng. J..

[26] Lotfipour F., Mirzaeei S., Maghsoodi M. (2012). Evaluation of the effect of CaCl_2_ and alginate concentrations and hardening time on the characteristics of *Lactobacillus acidophilus* loaded alginate beads using response surface analysis. Adv. Pharm. Bull..

[27] Liu J., Liu F., Ren T., Wang J., Yang M., Yao Y., Chen H. (2021). Fabrication of fish gelatin/sodium alginate double network gels for encapsulation of probiotics. J. Sci. Food Agric..

[28] Premjit Y., Pandey S., Mitra J. (2024). Encapsulation of probiotics in freeze-dried calcium alginate and κ-carrageenan beads using definitive screening design: A comprehensive characterisation and in vitro digestion study. Int. J. Biol. Macromol..

[29] Vimon S., Kertsomboon T., Chirachanchai S., Angkanaporn K., Nuengjamnong C. (2023). Matrices-charges of agar-alginate crosslinked microcapsules via o/w microemulsion: A non-spore forming probiotic bacteria encapsulation system for extensive viability. Carbohydr. Polym..

[30] Li J.M., Nie S.P. (2016). The functional and nutritional aspects of hydrocolloids in foods. Food Hydrocoll..

[31] Ramdhan T., Ching S.H., Prakash S., Bhandari B. (2020). Physical and mechanical properties of alginate based composite gels. Trends Food Sci..

[32] Ahmed E.M. (2015). Hydrogel: Preparation, characterization, and applications: A review. J. Adv. Res..

[33] Yan J., Zhang Z., Lai B., Wang C., Wu H. (2024). Recent advances in marine-derived protein/polysaccharide hydrogels: Classification, fabrication, characterization, mechanism and food applications. Trends Food Sci..

[34] Baybaş D., Serdaroğlu G., Semerci B. (2021). The composite microbeads of alginate, carrageenan, gelatin, and poly (lactic-co-glycolic acid): Synthesis, characterization and density functional theory calculations. Int. J. Biol. Macromol..

[35] Li Q., Shi J., Liu L., McClements D.J., Duan M., Chen X., Liu J. (2021). Encapsulation of fruit peel proanthocyanidins in biopolymer microgels: Relationship between structural characteristics and encapsulation/release properties. Food Hydrocoll..

[36] Wu Y., Lv B., Wang S., Liu Z., Chen X.D., Cheng Y. (2024). Study of molecular interaction and texture characteristics of hydrocolloid-mixed alginate microspheres: As a shell to encapsulate multiphase oil cores. Carbohydr. Polym..

[37] Abu Bakar M.H., Azeman N.H., Mobarak N.N., Mokhtar M.H.H., A Bakar A.A. (2020). Effect of active site modification towards performance enhancement in biopolymer κ-Carrageenan derivatives. Polymers.

[38] Jabli M., Almalki S.G., Agougui H. (2020). An insight into methylene blue adsorption characteristics onto functionalized alginate bio-polymer gel beads with λ-carrageenan-calcium phosphate, carboxymethyl cellulose, and celite 545. Int. J. Biol. Macromol..

[39] Chen H., Chen F., Xiao Q., Cai M., Yang Q., Weng H., Xiao A. (2021). Structure and physicochemical properties of amphiphilic agar modified with octenyl succinic anhydride. Carbohydr. Polym..

[40] Zhu W., Lyu F., Naumovski N., Ajlouni S., Ranadheera C.S. (2020). Functional efficacy of probiotic *Lactobacillus sanfranciscensis* in apple, orange and tomato juices with special reference to storage stability and in vitro gastrointestinal survival. Beverages.

[41] Liang J.R., Deng H., Hu C.Y., Zhao P.T., Meng Y.H. (2022). Vitality, fermentation, aroma profile, and digestive tolerance of the newly selected *Lactiplantibacillus plantarum* and *Lacticaseibacillus paracasei* in fermented apple juice. Front. Nutr..

[42] Castro-López C., Romero-Luna H.E., García H.S., Vallejo-Cordoba B., González-Córdova A.F., Hernández-Mendoza A. (2023). Key stress response mechanisms of probiotics during their journey through the digestive system: A review. Probiotics Antimicrob. Proteins.

[43] Wu Y., Geng F., Chang P.R., Yu J., Ma X. (2009). Effect of agar on the microstructure and performance of potato starch film. Carbohydr. Polym..

[44] Qi X., Simsek S., Chen B., Rao J. (2020). Alginate-based double-network hydrogel improves the viability of encapsulated probiotics during simulated sequential gastrointestinal digestion: Effect of biopolymer type and concentrations. Int. J. Biol. Macromol..

[45] Cook M.T., Tzortzis G., Charalampopoulos D., Khutoryanskiy V.V. (2012). Microencapsulation of probiotics for gastrointestinal delivery. J. Control Release.

[46] de Souza E.L., de Albuquerque T.M.R., Dos Santos A.S., Massa N.M.L., de Brito Alves J.L. (2019). Potential interactions among phenolic compounds and probiotics for mutual boosting of their health-promoting properties and food functionalities—A review. Crit. Rev. Food Sci. Nutr..

[47] Kumar B.V., Vijayendra S.V.N., Reddy O.V.S. (2015). Trends in dairy and non-dairy probiotic products—A review. J. Food Sci. Technol..

[48] Ni F., Luo X., Zhao Z., Yuan J., Song Y., Liu C., Huang M., Dong L., Xie H., Cai L. (2023). Enhancing viability of *Lactobacillus plantarum* encapsulated by alginate-gelatin hydrogel beads during gastrointestinal digestion, storage and in the mimic beverage systems. Int. J. Biol. Macromol..

[49] Wojtunik-Kulesza K., Oniszczuk A., Oniszczuk T., Combrzyński M., Nowakowska D., Matwijczuk A. (2020). Influence of in vitro digestion on composition, bioaccessibility and antioxidant activity of food polyphenols—A non-systematic review. Nutrients.

[50] Diez-Sánchez E., Quiles A., Hernando I. (2021). Interactions between blackcurrant polyphenols and food macronutrients in model systems: In vitro digestion studies. Foods.

[51] Meng Y., Hao L., Tan Y., Yang Y., Liu L., Li C., Du P. (2021). Noncovalent interaction of cyanidin-3-O-glucoside with whey protein isolate and β-lactoglobulin: Focus on fluorescence quenching and antioxidant properties. LWT-Food Sci. Technol..

